# Robust Orthogonal-View 2-D/3-D Rigid Registration for Minimally Invasive Surgery

**DOI:** 10.3390/mi12070844

**Published:** 2021-07-20

**Authors:** Zhou An, Honghai Ma, Lilu Liu, Yue Wang, Haojian Lu, Chunlin Zhou, Rong Xiong, Jian Hu

**Affiliations:** 1 Department of Thoracic Surgery, The First Affiliated Hospital, Zhejiang University School of Medicine, Hangzhou 310003, China; az-surgeon@hotmail.com (Z.A.); mhbjq123@163.com (H.M.); 2 State Key Laboratory of Industrial Control and Technology, Department of Control Science and Engineering, Zhejiang University, Hangzhou 310027, China; liluliu@zju.edu.cn (L.L.); wangyue@iipc.zju.edu.cn (Y.W.); luhaojian@zju.edu.cn (H.L.); c_zhou@zju.edu.cn (C.Z.); rxiong@zju.edu.cn (R.X.)

**Keywords:** 2-D/3-D registration, rigid, multi-view, reconstruction, deep learning

## Abstract

Intra-operative target pose estimation is fundamental in minimally invasive surgery (MIS) to guiding surgical robots. This task can be fulfilled by the 2-D/3-D rigid registration, which aligns the anatomical structures between intra-operative 2-D fluoroscopy and the pre-operative 3-D computed tomography (CT) with annotated target information. Although this technique has been researched for decades, it is still challenging to achieve accuracy, robustness and efficiency simultaneously. In this paper, a novel orthogonal-view 2-D/3-D rigid registration framework is proposed which combines the dense reconstruction based on deep learning and the GPU-accelerated 3-D/3-D rigid registration. First, we employ the X2CT-GAN to reconstruct a target CT from two orthogonal fluoroscopy images. After that, the generated target CT and pre-operative CT are input into the 3-D/3-D rigid registration part, which potentially needs a few iterations to converge the global optima. For further efficiency improvement, we make the 3-D/3-D registration algorithm parallel and apply a GPU to accelerate this part. For evaluation, a novel tool is employed to preprocess the public head CT dataset CQ500 and a CT-DRR dataset is presented as the benchmark. The proposed method achieves 1.65 ± 1.41 mm in mean target registration error(mTRE), 20% in the gross failure rate(GFR) and 1.8 s in running time. Our method outperforms the state-of-the-art methods in most test cases. It is promising to apply the proposed method in localization and nano manipulation of micro surgical robot for highly precise MIS.

## 1. Introduction

The surgical guidance system can be used to assist surgical robots in localizing manually annotated markers and anatomical structures concerning interventional instruments so that the surgical injury to normal tissue can be reduced [1]. For minimally invasive surgery (MIS), the nanomanipulation accuracy of micro robots also depends on the precision of the guidance system. In clinical scenarios, intra-operative 2-D fluoroscopy is commonly applied due to flexibility and efficiency. While some important structures (e.g., blood vessel [2] or heart [3]) are blurred and ambiguous in fluoroscopy as a result of dimensional reduction. Thus, pre-operative 3-D images (e.g., computed tomography (CT), cone-beam computed tomography (CBCT) or magnetic resonance imaging (MRI)) are considered to augment the 2-D images. 2-D/3-D rigid registration is the key enabling technology to implement the augmentation, which is widely researched for decades and is comprehensively reviewed by Markelj et al. and Liao et al. in [4,5]. This technology transforms 3-D images with 6 Degree-of-Freedom (DoF) to the coordinate system where 2-D images are projected, and align the anatomical structures between 2-D images and projections of 3-D images. To ensure high registration accuracy and robustness, multiple views of fluoroscopy are employed to avoid the ill-posed nature of single-view registration [6], leading to increased running time. Although many methods were proposed to improve the performance of 2-D/3-D rigid registration, trade-offs always have to be made among accuracy, robustness and efficiency. Referring to [7], existing 2-D/3-D rigid registration methods can be classified as optimization-based and learning-based methods.

For optimization-based approaches, the 2-D/3-D rigid registration is commonly modeled as an optimization problem. A digitally reconstructed radiograph (DRR) is rendered from a 3-D image by the Ray-casting algorithm [8] in the current pose. Then the intensity-based similarity between DRR and fluoroscopy is calculated as the objective function. Optimizers (e.g., CMA-ES and BFGS) with optimization strategies (e.g., multi-starts with local re-start and image pyramid) are employed to solve the globally optimal solution of 6 DoF pose. Due to the high non-convexity of similarity metrics (NGI, GS, GC, GO [9]) and the ill-posed nature of the single-view condition, the optimizer needs a huge number of iterations to find global optima. Thanks to the hardware-based acceleration [10], the speed of each iteration significantly improves. Multiple views of fluoroscopy images are used to enhance the robustness and accuracy, while it also increases running time for a successful registration. Chen et al. [11] extracted edges of anatomical structures in 2-D images. Virtual rays are formed by connecting 2-D points on edges to an X-ray source. Registration is performed by minimizing the distance between virtual rays and closed outer surface of the 3-D image. Tomazevic et al. [12] proposed a reconstruction-based approach that integrated multiple views of fluoroscopy to reconstruct a 3-D image. Then the best possible spatial correspondence between reconstructed and pre-operative 3-D images was solved by optimizing a novel similarity measure. This is the first work that brings the reconstruction method into the 2-D/3-D rigid registration task. To improve the registration accuracy, Markelj et al. [13] extracted gradients of both 2-D and 3-D images and a coarse 3-D gradient volume was reconstructed by several 2-D gradient maps. The registration was completed by matching the pre-operative and reconstructed gradient volumes. Although much effort has been made to improve the efficiency, the overall registration time is still non-negligible. Moreover, the above methods use low-level features of the image (e.g., intensity and gradient) to perform registration, which is sensitive to image artifacts and implanted instruments. In addition, optimization-based approaches are highly dependent on the initial pose, which introduces manual registration and increases the workload of clinicians.

For learning-based approaches, high-level features of the image are extracted by the network, thus the registration accuracy and robustness are potentially improved. Moreover, the efficiency of these methods is generally higher than optimization-based ones due to fewer iterations for convergence (or no need for iteration). Miao et al. [14] first applied a deep neural network (DNN) to the 2-D/3-D rigid registration task, in which the 6 DoF pose of the 3-D image was directly regressed using a pair of DRR and fluoroscopy image as input. For fast convergence, the 6 DoF pose was divided into three groups and estimated hierarchically. This work aimed at implanted instruments and single-view registration, and significantly outperformed optimization-based methods. For multi-view registration, Miao et al. [7] introduced a multi-agent system to solve the problem. Each agent was trained with a dilated fully convolutional network (FCN). The registration was performed in a Markov Decision Process (MDP) by observing a local region. Results on the spine CBCT dataset showed that the MDP-based method achieved the best performance compared to the state-of-the-art optimization-based approaches. While it is inevitable that the local search might reach an unseen pose and make registration fail. Liao et al. [15] proposed a tracking-based method to make full use of image information and use the strength of the landmark-based method. Some 3-D points of interest (POIs) were randomly chosen in 3-D images and projected into DRRs in two orthogonal views. Then a siamese network was employed to track the same 2-D POIs in both DRRs and fluoroscopy images. After that, the tracked 2-D POIs in fluoroscopy images were reconstructed to 3-D by triangulation. The registration problem was converted to the matching of two 3-D point sets, which could be solved by the iterative closest point (ICP) algorithm. This work uses sparse points for reconstruction and registration, which concentrate on the tracking of 2-D POIs using local appearance similarity. Thus, some simple but important information may be omitted in registration and the highly repetitive structures (e.g., ribs and vertebrae) may cause performance reduction.

**Figure 1:**
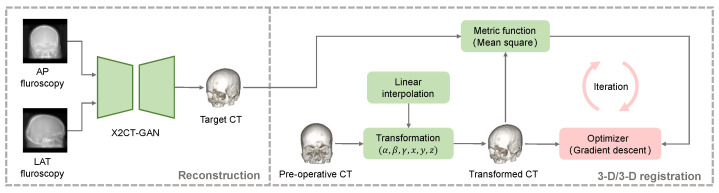
The framework of the proposed method: In the reconstruction stage, two orthogonal views of fluoroscopy images are input into the X2CT-GAN to reconstruct the target CT image. In the 3-D/3-D registration stage, the pre-operative CT is aligned with the target CT using a GPU-accelerated optimization framework. Green patches in the illustration denote parts implemented by GPU, and red patch denotes the part run on CPU.

In this paper, we propose a novel orthogonal-view 2-D/3-D rigid registration framework which integrates the deep-learning-based dense reconstruction with the GPU- accelerated 3-D/3-D registration, shown as Figure 1. In the reconstruction stage, we follow the literature [16] to implement the dense reconstruction of a pseudo-CT as the target CT from two orthogonal views of radiographs. The dense reconstruction avoids the ill-posed nature and dimensional reduction of direct 2-D/3-D registration. Thus, this problem is converted to the registration of two images with the same dimension (3-D), which potentially reduces the difficulty of optimization. In the 3-D/3-D registration stage, the pre-operative CT image is used as the moving image and a GPU-based fast 3-D/3-D registration method is proposed for aligning the target and moving CT images. The main contributions of our work are summarized as follows.

- We convert the traditional orthogonal-view 2-D/3-D registration problem into the 3-D/3-D registration problem by leveraging a deep-learning-based dense reconstruction network for achieving high registration accuracy and robustness.
- We introduce the parallelization strategy and use GPU to accelerate the 3-D/3-D registration for achieving desired performance in an acceptable period of time.
- We apply the 2-D/3-D registration technique in radiofrequency ablation (RFA) of the treatment of trigeminal neuralgia for the first time and the proposed method achieves the better performance among existing methods on this task.

## 2. Materials and Methods

### 2.1. Problem Description

**Figure 2:**
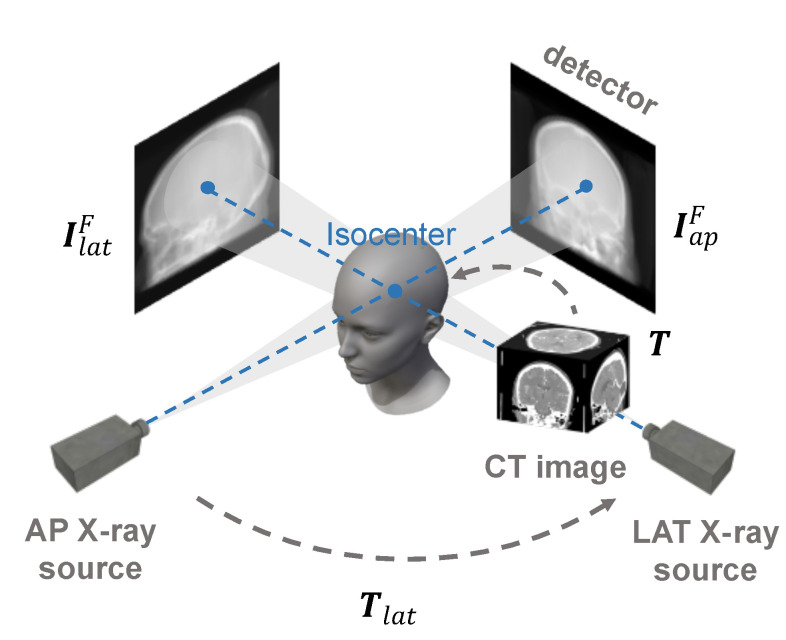
The X-ray imaging model of mult-view 2-D/3-D rigid registration. The goal of registration is to solve the transformation T.

For multi-view 2-D/3-D rigid registration, we assume that the two 2-D images are orthogonal head fluoroscopy images acquired in anterior-posterior (AP) and lateral (LAT) view respectively, and the 3-D image is head CT. Without loss of generality, we suppose that the X-ray imaging system is a pinhole camera model and the system is well calibrated, shown in Figure 2. Thus, the projection $I^{P}:R^{2}\to R$ of CT image $J:R^{3}\to R$ on the detector can be defined as

(1) $$I^{P}\left(x;T\right)=\int_{p\in L(x,r)}J(T^{-1}p)dp$$

where x is a 2-D point on detector, and L(x,r) is a virtual ray that link the X-ray source and x, and *r* denotes the parameter of the virtual ray where the 3-D point p lies on. The 6 DoF transformation (i.e., pose) T brings CT from its own coordinate system to the patient’s one. T can be parameterized as three rotation angles, $\theta ={\left(\alpha ,\beta ,\gamma \right)}^{T}$, and three translation, $t={\left(t_{x},t_{y},t_{z}\right)}^{T}$, about axes. Also, T can be written as 4 × 4 matrix as

(2) $$T=\left[\begin{array}{cc} R(\theta ) & t\\ 0 & 1 \end{array}\right]\in R^{4\times 4}$$

where $R\left(\theta \right)\in R^{3\times 3}$ denotes the rotation matrix. The objective of multi-view 2-D/3-D rigid registration is to find the best pose that maximizes the similarity between projections $I^{P}$ and real fluoroscopy images $I^{F}$ as

(3) $$T^{*}=\underset{T}{\arg\max}\;\sum_{i=1}^{2}F\left(I_{i}^{F},I_{i}^{P}\left(T\right)\right)$$

where $i\in \left\{1,2\right\}$ denotes the AP and LAT view respectively, and F(·) represents the similarity metric of 2-D images. For the AP view, the projection can be formulated as Equation (1). As there is a fixed transformation between different views, the lateral projection can be defined as

(4) $$I_{lat}^{P}\left(x;T\right)=\int_{p\in L(x,r)}J(T^{-1}T_{lat}^{-1}p)dp$$

In practice, the projection from CT, known as digitally reconstructed radiograph (DRR) can be implemented by the Ray-casting algorithm. In this paper, rather than directly optimize the highly non-convex and time-consuming problem as Equation (3), we firstly reconstruct a targe 3-D image from two views of fluoroscopy as

(5) $$J_{target}=R\left(I_{1}^{F},I_{2}^{F}\right)$$

where R(·) denotes the reconstruction function. Then the 2-D/3-D registration can be equivalently performed by 3-D/3-D registration between pre-operative and target CT as

(6) $$T^{*}=\underset{T}{\arg\max}G\left(J\left(T\right),J_{target}\right)$$

where G(·) is the similarity metric of 3-D images. Compared with Equation (3), Equation (6) optimize the pose of the 3-D image using the same dimension metric, which avoids the ill-posed condition and rendering process, and can converge to global optima using a small number of iterations in theory.

### 2.2. Dense Reconstruction Model

Before 2-D/3-D rigid registration, the correspondence between 2-D and 3-D space needs to be established. Instead of sparse reconstruction such as [15], we densely reconstruct a new CT volume, name as target CT, from two orthogonal fluoroscopy images to make full use of information in pre- and intra-operative images for registration.

3-D model reconstruction from 2-D projections has been researched for decades [17,18,19], and most of the existing methods reconstruct the outer surface of 3-D models due to the opaqueness to light. While X-ray can penetrate most structures of human and fluoroscopy image contains much anatomical information, which can be used to reconstruct 3-D organs such as CT volume. Traditional CT reconstruction methods [20] need a large number of fluoroscopy images in different views, which consume much time in image acquisition and reconstruction. Henzler et al. [21] first employed deep learning to reconstruct a CT volume from a single 2-D X-ray image. However, a single view image led to much ambiguity due to the loss of depth information. Ying et al. [16] designed an encoder-decoder framework to reconstruct CT volume from two orthogonal 2-D X-ray images and integrated it into an adversarial training process, named X2CT-GAN. The reconstruction accuracy was significantly improved compared [21]. In this paper, we adopt a similar architecture for dense reconstruction from [16].

According to the generator architecture of [16], two parallel encoder-decoder networks are designed to learn the mapping from two views of 2-D to 3-D images in the feature space. In addition, the fusion network is responsible to integrate the information of two encoder-decoder networks for generating the 3-D CT. Referring to [22], the encoder includes a series of dense modules with spatial down-sampling. The decoder consists of basic 3-D convolution blocks and is linked with the encoder using a fully connected layer and some skip connections. Then the extracted biplanar features are fused by a concatenation of convolution blocks which is similar to the decoder. The structure of the discriminator is based on CNN, called 3DPatchDiscriminator [23]. In deep network architectures, the activation function should be chosen carefully since they have an important role in performance [24]. Therefore, we used ReLU due to its efficiency in our network architecture.

### 2.3. Fast 3-D/3-D Registration

When the reconstruction is completed, we perform 3-D/3-D registration using pre-operative and reconstructed CT. Due to the loss of depth information, direct 2-D/3-D registration in each view is an ill-posed problem. It is suitable to use global gradient-free optimizers for solving the 6 DoF pose of CT with 2-D images’ similarity measured. These optimizers need a huge number of iterations to find global optima and the rendering process is necessary for each iteration. To reduce the computational cost, 3-D/3-D registration with gradient-based optimization is employed. There is no dimensional reduction in 3-D images’ similarity metric and the optimizer can converge faster with fewer iterations than direct way.

The framework of 3-D/3-D registration is shown in Figure 1 The pre-operative CT is considered to be moving CT and the reconstructed CT is fixed CT. All CT images are in the same coordinate system. When the current transformation is applied on moving CT, a metric function measures the similarity between fixed and transformed CT images. In this paper, we calculate the mean square error (MSE) function to reflect the similarity as:

(7) $$MSE\left(J^{T},J^{F}\right)=\frac{1}{N}\sum_{i=1}^{N}{\left[J^{T}\left(i\right)-J^{F}\left(i\right)\right]}^{2}$$

where J(i) is the *i*-th voxel of 3-D image J, and *N* is the number of voxels considered. Minimizing the MSE function is equivalent to maximizing the similarity of 3-D images. Then the gradient descent algorithm is introduced to update transformation parameters. As the transformation is continuous and the CT image is discrete, there is a need to interpolate for transformed CT voxels. In addition, the linear interpolation is performed in this work.

Although fewer iterations are needed in 3-D/3-D registration than 2-D/3-D one, the transformation of 3-D image and similarity measuring require many computational resources. For further acceleration, we use GPU to calculate these simple and time-consuming parts in parallel.

### 2.4. Loss Design and Training Strategy

For reconstruction of target CT, we employ X2CT-GAN to generate a 3-D image from biplanar fluoroscopy images. The adversarial training process of X2CT-GAN can be divided into generator part and discriminator part. As the conditional LSGAN is proved to have the best performance in [16], the loss function of the discriminator can be defined as

(8) $$L_{LSGAN}\left(D\right)=\frac{1}{2}\left[E_{y∼p(CT)}{\left(D\left(y|x\right)-1\right)}^{2}+E_{x∼p(fluoroscopy)}{\left(D\left(G\left(x\right)|x\right)-0\right)}^{2}\right]$$

where *x* denotes the pair of input biplanar fluoroscopy images that subjects the distribution p(fluoroscopy), and *y* is the ground truth CT image that subjects to p(CT). The loss function of the generator can be defined as

(9) $$L_{LSGAN}\left(G\right)=\frac{1}{2}\left[E_{x∼p(fluoroscopy)}{\left(D\left(G\left(x\right)|x\right)-1\right)}^{2}\right]$$

To provide higher precision of internal 3-D structures, the reconstruction loss function is combined with generator loss using MSE as

(10) $$L_{rl}=E_{x,y}{∥y-G(x)∥}_{2}^{2}$$

In the original X2CT-GAN, the authors proposed a projection loss function based on the orthogonal projection for supervision. While in clinical scenarios, fluoroscopy is commonly performed by the C-arm system, which can be modeled as a pinhole camera with the perspective projection [25]. Thus, we introduce a perspective projection loss function to simulate the real clinical setting for better supervision. In this function, the projections of pseudo-CT are rendered by the Ray-Casting algorithm and the MSE between input X-rays and new projections is calculated as:

(11) $$L_{rpl}=\frac{1}{2}\left[E_{x,y}{∥I_{ap}^{F}-P_{ap}\left(G\left(x\right)\right)∥}_{2}^{2}+E_{x,y}{∥I_{lat}^{F}-P_{lat}\left(G\left(x\right)\right)∥}_{2}^{2}\right]$$

where $P_{ap}\left(\cdot \right)$ and $P_{lat}\left(\cdot \right)$ denote the Ray-casting function of AP and LAT view respectively. Then we make the Ray-casting algorithm differentiable so that the loss can be back-propagated to train our network.

The final loss function of discriminator $L_{D}$ is equal to $\epsilon_{1}L_{LSGAN}\left(D\right)$ and the total loss function of generator is defined as the combination of the above loss:

(12) $$L_{G}=\epsilon_{1}L_{LSGAN}\left(G\right)+\epsilon_{2}L_{rl}+\epsilon_{3}L_{rpl}$$

where $\epsilon_{1}$, $\epsilon_{2}$ and $\epsilon_{3}$ are balance parameters of different loss terms.In this paper, we set $\epsilon_{1}$, $\epsilon_{2}$ and $\epsilon_{3}$ to 0.1, 10 and 10 respectively. In addition, the reconstruction network is trained for 100 epochs. The learning rate of the Adam solver is 2 $\times \;10^{-4}$. The ratio of training and test set is 4:1.

## 3. Experimental Results

### 3.1. Dataset

To demonstrate the performance of the proposed method, we aim at the radiofrequency ablation(RFA) in trigeminal neuralgia. The 2-D/3-D rigid registration can be used to localize foramen ovales under radiographs. In clinical scenarios, the RFA is commonly guided by C-arms. Clinicians need to find the foramen ovales in radiographs by human eyes for precise puncture [26]. While it is difficult for clinicians to find an appropriate orientation for imaging and discriminate the foramen ovales under radiographs with many complicated bone structures overlapping. Therefore, 2-D/3-D registration is employed for foramen ovales localization. Two foramen ovales are annotated by clinicians in pre-operative CT images. During the operation, pre-operative CT is automatically aligned with intra-operative radiographs using the proposed method. It is an effective way to assist clinicians to find the target foramen ovales for puncture.

**Figure 3:**
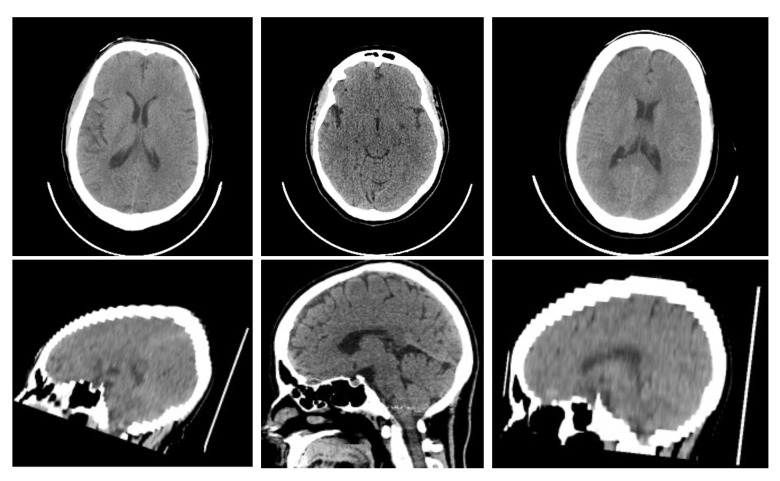
Three examples of head CTs in the CQ500 dataset.

**Figure 4:**
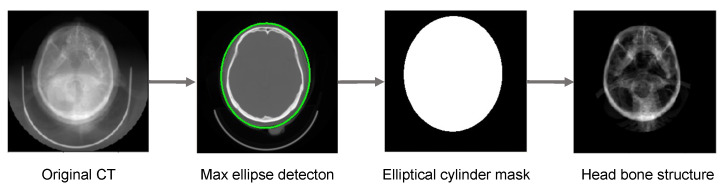
The workflow of the proposed preprocessing tool. The elliptical mask is 20% greater than the max detected ellipse.

For evaluating the proposed method, we use a public head CT dataset CQ500 [27], which contains anonymized dicoms of 1269 CT scans for 491 patients and the corresponding radiologists’ reads. Some examples of CQ500 are shown in Figure 3 Based on CQ500, we introduce a CT-DRR dataset with augmentation. Specifically, two DRRs are rendered from the original CT image in AP and LAT view respectively. It is an established way that using DRR as fluoroscopy to evaluate 2-D/3-D registration methods [28]. In addition, the calibration error can be eliminated by this hypothesis. As the DRR is rendered in a specific view, the ground-truth transformation of 2-D/3-D registration between the CT and DRR can be considered to be the rendering view. In clinical scenarios, the patient’s head is commonly laid on the metal bed so that there is much unrelated content scanned by the CT machine. Thus, we process the original CT image to remove this useless bed information as Figure 4. Considering the image features of CT, we first detect the max ellipse among all slices and expand it to 120% the original size. Then we crop the CT volume with the corresponding elliptical cylinder so that only head is reserved in the CT image. For performing rigid registration, we remove these sparse tissues whose HU < 100 in CT image and leave the highly rigid bone structure. After that, a sample of the CT-DRR dataset is completed. Moreover, we randomly shift the view of X-ray imaging around the initial pose for data augmentation. The range of shifting is (−10, 10${}^{\circ }$) for rotation and (−20, 20 mm) for translation.

For training the proposed method, 75% samples of the above dataset are randomly chosen as a training set. In the left dataset, ten CT images are selected and projected for rendering corresponding DRRs in an extreme pose (i.e., 10${}^{\circ }$ for rotation and 20 mm for translation). The newly generated CT-DRR dataset is used as a test set.

### 3.2. Metrics

**Figure 5:**
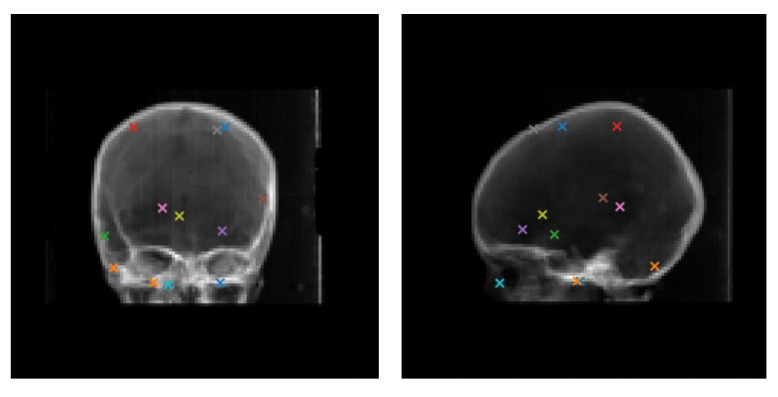
A example of 12 landmarks in head CT image.

Referring to [29], the accuracy of 2-D/3-D rigid registration methods can be evaluated with a standard metric named mean target registration error (mTRE). This metric measures the mean distance between the ground-truth CT landmarks and the aligned CT landmarks in 3-D space:

(13) $$mTRE=\frac{1}{N}\sum_{i=1}^{N}{∥T_{reg}p_{i}-T_{gt}p_{i}∥}_{2}$$

where *N* is the number of landmarks p, and $T_{reg}$ is the transformation (or pose) result of 2-D/3-D registration method, and $T_{gt}$ is the ground-truth transformation. In this paper, the landmarks in the target region are two central points of foramen ovale (FO) annotated and checked by several experienced clinicians in every head CT of the test set. In addition, we randomly choose other ten landmarks for evaluation in the range of whole bone structures in head CT, shown in Figure 5. In this way, both target and other regions are taken into consideration. Moreover, we report the gross failure rate(GFR) and average running time for evaluating the robustness and efficiency of 2-D/3-D rigid registration methods. The failure criterion is defined as mTRE > 3 mm [30] for radiofrequency ablation of trigeminal neuralgia.

### 3.3. Results

**Figure 6:**
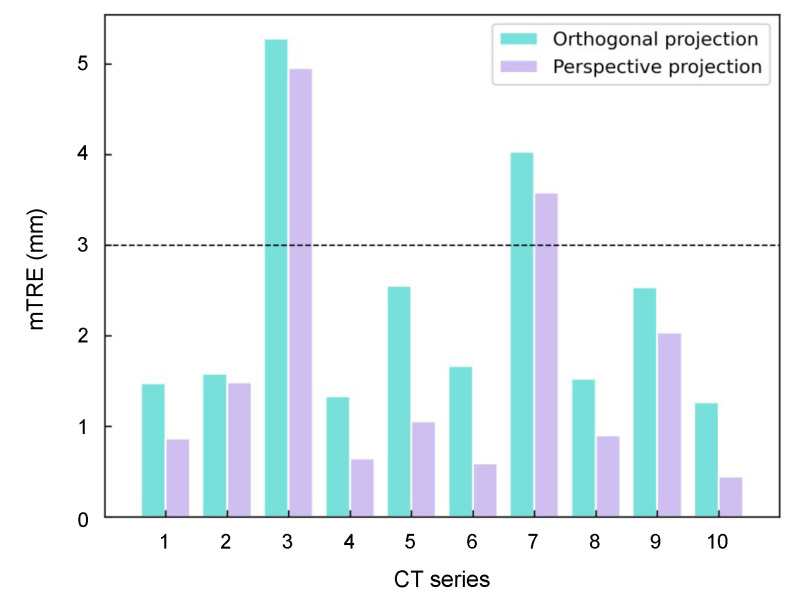
The mTRE of different projection loss functions.

To demonstrate the effectiveness of perspective projection loss function, an ablation study is conducted with the same experimental setup except for the projection term of the loss function. As the result showing in Figure 6, the network supervised by the perspective projection loss leads to slightly better registration accuracy than that supervised by the orthogonal projection loss.

For comparison, we implement the state-of-the-art 2-D/3-D registration approach POINT${}^{2}$ [15], which can also be used as an initial pose estimator for optimization-based methods [9,31] called POINT${}^{2}$+opt. We randomly choose 20 3-D points in each CT image as POIs and train the network with the same learning parameters as [15]. Additionally, we implement a commonly used optimization-based approach Opt-NGI [31].

**Figure 7:**
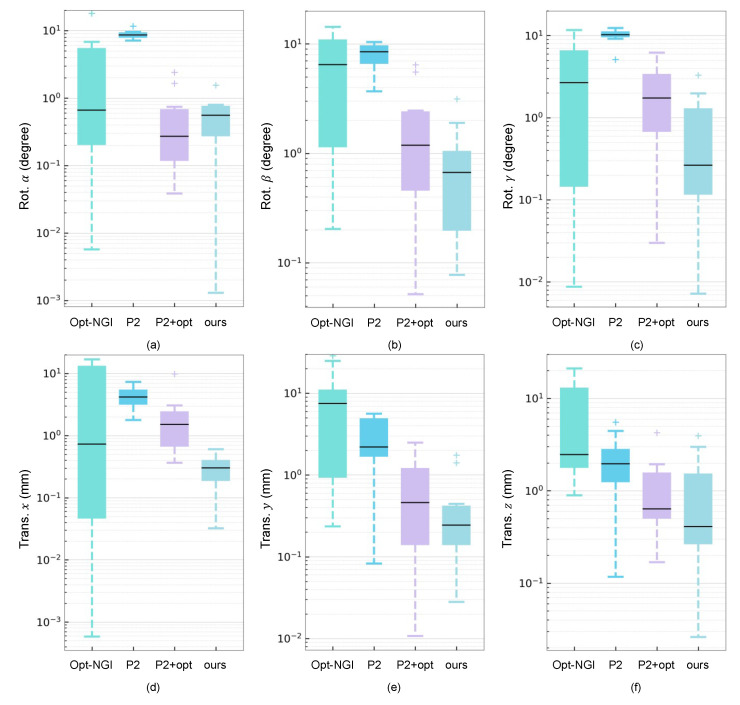
The distribution of errors in 6 DoF of four methods, including rotation errors in (**a**) *α*, (**b**) *β*, (**c**) *γ* and translation errors in (**d**) *x*, (**e**) *y*, (**f**) *z*.

The specific accuracy results of different methods on the CT-DRR dataset are summarized in Table 1 and Figure 7, where P2 denotes POINT${}^{2}$. In most cases, the proposed method achieves the lowest mTRE, demonstrating the highest comprehensive accuracy. As for 6 DoF pose estimation, the proposed method outperforms other methods in rotation β, γ, and translation *x*, *y*. The errors of our method in rotation α and translation *z* are comparable to those of POINT${}^{2}$+opt. As AP and LAT views are along axis *x* and *y* respectively, β and γ can be seen as in-plane parameters in each view, which is inherently accurate and easy to estimate. Thus, the out-of-plane parameter α has the largest rotation error and the translation *z* is correspondingly affected. In summary, the proposed method obviously outperforms Opt-NGI and POINT${}^{2}$ in comprehensive accuracy and is better than POINT${}^{2}$+opt in most metrics of accuracy. As for Opt-NGI, the results have a large variance in all DoF of the pose. The optimization-based method is sensitive to the initial pose and always needs a long time for searching the global optima. Moreover, an evolution strategy is employed for random starts of searching, which may cause a large variance of registration results. Although Opt-NGI can achieve relatively high accuracy in certain DoF of some cases, the comprehensive accuracy (i.e., mTRE) is still lower than the proposed method in all test cases. For POINT${}^{2}$, the sparse POIs tracking strategy introduces local ambiguity when target fluoroscopy and initial DRR are generated in quite different views. For example, a 3-D point of the skull in CT is projected to DRR in initial view and the formed 2-D point is located on the edge of the head in DRR. However, the target view when generating the fluoroscopy is possibly quite far from the initial view and the target 2-D projected point may not be located on the edge. The POINT${}^{2}$ tracks the most similar structure between fluoroscopy and DRR so that the inherent misalignment is introduced. While our method directly performs dense reconstruction from biplanar DRRs, which concentrates global and high-level features of anatomical structures and avoids local mismatches. As for POINT${}^{2}$+opt, an optimization-based algorithm is used to refine the result of POINT${}^{2}$. In theory, the optimization time is positively correlated with the distance between the initial pose and the ground truth pose. The worse initialization POINT${}^{2}$ provides, the more optimization time is needed. However, the running time is limited in clinical scenarios, so the trade-off between the efficiency and accuracy of registration is necessary. Referring to [15], we set optimization parameters to limit the whole time of POINT${}^{2}$+opt within 3 s. Thus, a few well-initialized cases can converge to global optima quickly. While most cases have poorer initialization from POINT${}^{2}$ and they still hold large errors after optimization-based pose refinement. THat is the reason POINT${}^{2}$+opt is competitive with the proposed method in some cases but not as good as ours in general.

**Figure 8:**
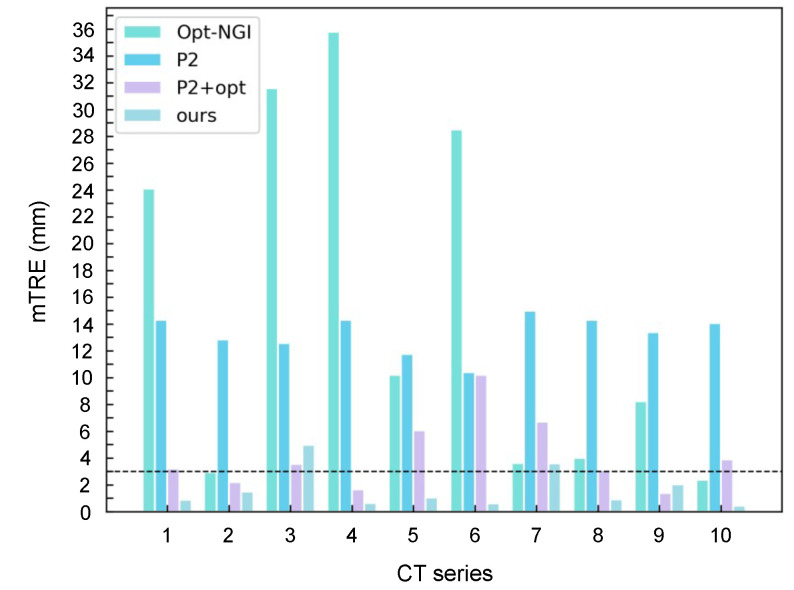
The mTRE of three methods for ten test cases. The criterion of a successful registration is mTRE < 3 mm.

The mTRE results of the three methods are illustrated in Figure 8. According to the above failure criterion, the GFR results of Opt-NGI, POINT${}^{2}$, POINT${}^{2}$+opt, and our method are 80%, 100%, 30%, and 20% respectively, demonstrating that the proposed method has the best robustness. Compared with POINT${}^{2}$+opt, which is designed as a coarse-to-fine algorithm, our method achieves better performance without applying any optimization-based 2-D/3-D registration method for pose refinement. This is mainly due to the abundant and accurate 3-D information provided by dense reconstruction.

**Figure 9:**
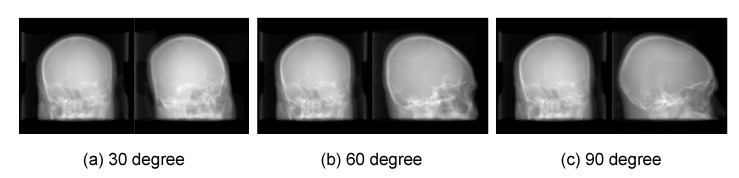
Examples of CT-DRR datasets in different projection angles.

**Figure 10:**
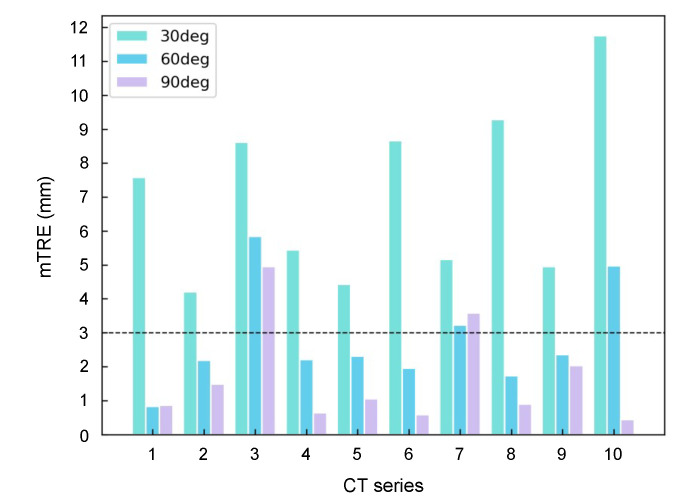
The mTRE of different angles.

For evaluating the effects of the angle between two views of X-ray on the registration accuracy. The angle is set to 30, 60 and 90 degrees respectively for three trails. For each trial, a CT-DRR dataset with a specific projection angle is generated using the Ray-casting algorithm, shown in Figure 9. As the evaluation on 90 degrees (i.e., orthogonal view) has been reported in the manuscript, we retrain the reconstruction network using CT-DRR datasets of 30 and 60 degrees respectively, with the training parameters same as the 90 degree trail. Then the 2-D/3-D registration is implemented on ten test cases. The mTRE of three trails are summarized in Figure 10. The results show that the 90 degree trail has the best accuracy. Because the orthogonal view can obtain more useful and non-repetitive information than other angles for reconstruction.

**Figure 11:**
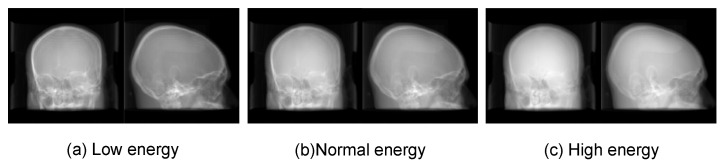
Examples of CT-DRR datasets in different X-ray energies.

**Figure 12:**
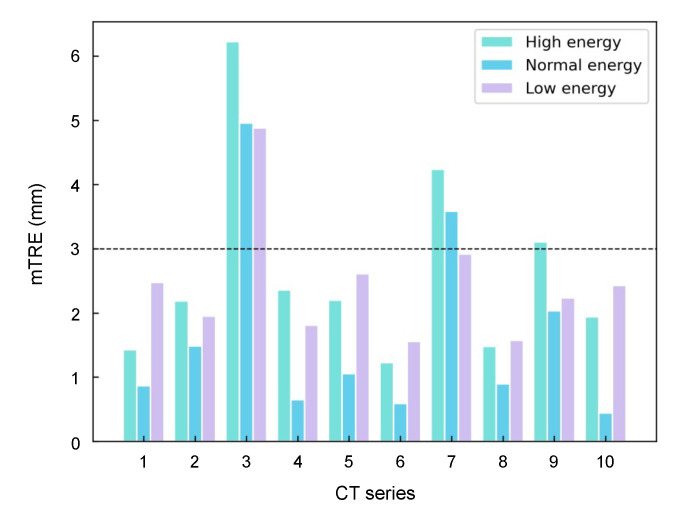
The mTRE of different energies.

For evaluating the effects of the different X-ray energies between fluoroscopy and CT on registration accuracy. Due to the limit of simulation, there is no quantitative relationship between DRR and CT image, but it is well-known that the contrast ratio of fluoroscopy image is negatively correlated with X-ray energy. Thus, we qualitatively vary the contrast ratio of DRR from low to high to simulate radiographs with different X-ray energies. Three varied CT-DRR datasets are generated in orthogonal view as Figure 11. Then the reconstruction network is retrained using these datasets with the same training parameters as the above experiments. After that, the 2-D/3-D registration is implemented on ten test cases. The mTRE of three trails are summarized in Figure 12. The result shows that high energy is not beneficial to improving registration accuracy. This is due to the bone structure, which is the key point of reconstruction and rigid registration, have low contrast with other tissue in the radiograph. But generally speaking, the effect of different X-ray energies between X-ray and CT influence on registration accuracy is not obvious.

The results of running time are shown in Table 2. The POINT${}^{2}$ consumes the shortest time for registration and our method has comparable efficiency with POINT${}^{2}$+opt. In our method, the reconstruction stage consumes about 0.6 s and the 3-D/3-D registration stage consumes about 1.2 s. Note our method does not need the optimization-based pose refinement as POINT${}^{2}$+opt and achieves better accuracy and robustness than other methods. Thus, it is a potential application that using our method as an initial pose estimator so that fewer iterations of optimization-based algorithm are needed because the initial pose solved by our method is closer to the global optima.

## 4. Discussion

**Figure 13:**
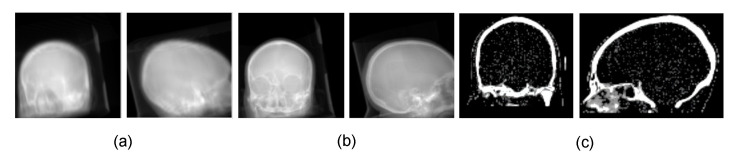
(**a**) DRRs of Case 3; (**b**) DRRs of Case 4; (**c**) CT image of Case 7. (**a**,**c**) are two failure cases using the proposed method. While (**b**) is a successful case.

For further discussion, we present two failure cases, Case 3 and Case 7, of the proposed method in Figure 13. In Case 3, the thickness between CT slices is 5 mm, which has low image resolution. So bone structures are indistinct in the DRR rendered from that CT, which intensely increases the difficulty of registration. In Case 7, much speckle-noise occurs in CT image due to the low quality of equipment. The noise has high HU values and is likely to be treated as the bone structure, which significantly affects the performance of registration. Moreover, we also present a successful case with high resolution and low noise as a contrast in Figure 13b. Therefore, we can see that low noise and high resolution of CT image are beneficial for improving registration accuracy.

Furthermore, we analyze the effects of the angle between two views of radiographs as well as the different X-ray energies between fluoroscopy and CT on the registration accuracy. According to the results shown in Figure 10, we can conclude that the orthogonal views of radiographs are able to provide the most sufficient information than other angles for reconstruction. Thus, in clinical practice, clinicians are suggested to perform fluoroscopy in two orthogonal views. According to the results shown in Figure 12, the effect of different X-ray energies between fluoroscopy and CT on registration accuracy is not obvious in the proposed method. Thus, it is possible for our work to fit different types of equipment in clinical scenarios.

Moreover, it is promising to improve the registration performance by image super-resolution. In clinical practice, high-resolution CT is commonly treated as a supplement to ordinary CT because of its higher dose of radiation and higher requirements for equipment. Image super-resolution techniques are able to increase the resolution of CT images without any extra radiation. By using high-resolution CT images, the accuracy and robustness of the proposed method can be significantly improved.

## 5. Conclusions

In this paper, a novel multi-view 2-D/3-D rigid registration method for minimally invasive surgery is proposed, which firstly densely reconstructs a target CT from biplanar fluoroscopy images and then performs a GPU-based 3-D/3-D rigid registration for final pose estimation. The dense reconstruction step preserves more useful information about the shape and posture of the patient than traditional projection-based and sparse-reconstruction-based approaches so that higher accuracy and robustness are achieved by our method. As the reconstructed image has the same dimension as the pre-operative image, the difficulty of optimization in 3-D/3-D registration is reduced and fewer iterations are needed for convergence than optimization-based 2-D/3-D registration. Thus, the efficiency of our method is potentially improved. The experimental results on the CT-DRR dataset show that our method achieves 1.65 ± 1.41 mm in mTRE, 20% in GFR and 1.8 s in running time. Our method outperforms the state-of-the-art approach POINT${}^{2}$+opt in most test cases. It is feasible to apply our method in tasks that need highly accurate poses in limited time, such as real-time navigation and nanomanipulation of micro robots for MIS.

## Figures and Tables

**Table 1:** Specific errors in 6 DoF and mTRE of three methods on the CT-DRR dataset.

CT Series	Methods	Rot. Error (${}^{\circ }$)			Trans. Error (mm)			mTRE↓
		*α*	*β*	*γ*	*x*	*y*	*z*	
#1	Opt-NGI	6.42	7.27	4.80	15.03	8.27	16.30	24.09
	P2	9.27	10.07	9.14	1.78	4.59	3.05	14.29
	P2+opt	0.09	2.21	0.73	3.07	0.02	0.53	3.21
	ours	0.74	0.67	0.34	0.49	0.27	0.45	0.86
#2	Opt-NGI	0.78	5.68	0.03	0.82	0.48	2.13	2.93
	P2	7.90	8.21	9.90	2.74	2.00	4.44	12.84
	P2+opt	0.12	0.29	0.03	2.07	0.01	0.59	2.18
	ours	0.43	1.14	1.43	0.03	0.03	0.70	1.48
#3	Opt-NGI	6.78	14.31	5.82	10.87	28.82	2.55	31.59
	P2	9.57	6.11	9.60	4.52	1.49	0.12	12.58
	P2+opt	2.41	1.29	0.67	0.37	2.50	1.94	3.56
	ours	0.67	3.14	3.29	0.19	1.75	3.93	4.96
#4	Opt-NGI	18.04	0.20	11.69	13.76	24.85	21.10	35.80
	P2	8.75	8.73	12.28	3.18	1.67	2.00	14.29
	P2+opt	0.04	1.07	2.29	1.55	0.48	0.50	1.65
	ours	0.79	0.10	0.19	0.33	0.12	0.12	0.64
#5	Opt-NGI	0.08	12.29	11.59	0.0006	8.34	1.68	10.18
	P2	7.55	6.25	10.61	3.86	0.08	0.51	11.74
	P2+opt	0.74	5.54	6.18	0.61	0.44	0.17	6.05
	ours	0.001	0.66	0.87	0.29	0.44	0.37	1.05
#6	Opt-NGI	0.18	1.31	0.46	16.83	11.77	19.68	28.48
	P2	9.17	3.68	5.10	4.94	5.63	1.94	10.39
	P2+opt	0.13	0.46	0.59	9.82	1.66	1.79	10.19
	ours	0.35	0.08	0.009	0.42	0.33	0.03	0.59
#7	Opt-NGI	2.40	1.10	0.04	0.001	2.21	2.68	3.62
	P2	11.63	10.42	9.95	3.29	2.40	5.51	14.95
	P2+opt	1.64	6.43	3.69	0.67	0.08	4.24	6.70
	ours	0.08	1.90	1.97	0.32	1.41	2.99	3.58
#8	Opt-NGI	0.28	9.23	0.009	0.12	0.52	0.89	4.00
	P2	8.51	8.88	12.40	7.32	4.98	2.05	14.28
	P2+opt	0.29	0.05	2.41	2.54	0.67	0.88	3.08
	ours	0.77	0.25	0.007	0.60	0.07	0.26	0.90
#9	Opt-NGI	0.006	11.42	6.76	0.65	6.74	1.07	8.22
	P2	7.14	9.83	11.06	5.55	5.61	1.12	13.37
	P2+opt	0.25	0.46	1.19	0.71	0.31	0.69	1.37
	ours	1.54	0.76	0.11	0.19	0.22	1.79	2.03
#10	Opt-NGI	0.54	0.39	0.55	0.02	0.23	2.38	2.37
	P2	8.32	7.76	11.10	6.18	1.78	1.65	14.05
	P2+opt	0.46	2.45	5.09	1.46	1.37	0.21	3.89
	ours	0.25	0.18	0.15	0.04	0.21	0.28	0.44

**Table 2:** The running time results of three methods. The proposed method has comparable efficiency with P2+opt.

Methods	Running Time(s)
Opt-NGI	18.1 ± 2.4
P2	0.7 ± 0.1
P2+opt	1.9 ± 0.8
ours	1.8 ± 0.3

## Data Availability

The data supporting reported results are available upon reasonable request.

## Abbreviations

The following abbreviations are used in this manuscript:

MIS	Minimally invasive surgery
CT	Computed tomography
CBCT	Cone beam computed tomography
MRI	Magnetic resonance imaging
DoF	Degree-of-Freedom
AP	Anterior-posterior
LAT	Lateral
DRR	Digitally reconstructed radiogragh
POI	Point of interest
MSE	Mean square error
mTRE	Mean target registration error
GFR	Gross failure rate
